# Acceptability and proof of concept of internet-delivered treatment for depression, anxiety, and stress in university students: protocol for an open feasibility trial

**DOI:** 10.1186/s40814-016-0068-9

**Published:** 2016-06-15

**Authors:** Patricia Frazier, Derek Richards, Jacqueline Mooney, Stefan G. Hofmann, Deborah Beidel, Patrick A. Palmieri, Christopher Bonner

**Affiliations:** 1 Department of Psychology, University of Minnesota, Minneapolis, USA; 2 SilverCloud Health, The Priory, John’s Street West, Dublin, Ireland; 3 School of Psychology, Trinity College Dublin, Dublin, Ireland; 4 Department of Psychological and Brain Sciences, Boston University, Boston, USA; 5 UCF RESTORES, Department of Psychology, University of Central Florida, Orlando, USA; 6 Center for the Treatment and Study of Traumatic Stress, Summa Health System, Akron, OH USA

**Keywords:** Depression, Anxiety, Stress, Online interventions, University students, CBT

## Abstract

**Background:**

In recent years, university counseling and mental health services have reported an increase in the number of clients seeking services and in yearly visits. This trend has been observed at many universities, indicating that behavioral and mental health issues pose significant problems for many college students.

The aim of this study is to assess the acceptability and proof of concept of internet-delivered treatment for depression, anxiety, and stress for university students.

**Methods/design:**

The study is an open feasibility trial of the SilverCloud programs for depression (Space from Depression), anxiety (Space from Anxiety), and stress (Space from Stress). All three are 8-module internet-delivered CBT (iCBT) intervention programs. Participants are assigned a supporter who provides weekly feedback on progress and exercises. Participants will complete the Patient Health Questionnaire-9 (PHQ-9), Generalized Anxiety Disorder-7 (GAD-7), and stress subscale of the Depression, Anxiety, Stress Scale-21 (DASS-21) as the outcome measures for the depression, anxiety, and stress interventions, respectively. Other outcomes include measures of acceptability of, and satisfaction, with the intervention. Data will be collected at baseline, 8 weeks and 3-month follow-up.

**Discussion:**

It is anticipated that the study will inform the researchers and service personnel of the programs’ potential to reduce depression, anxiety, and stress in a student population as well as the protocols to be employed in a future trial. In addition, it will provide insight into students’ engagement with the programs, their user experience, and their satisfaction with the online delivery format.

## Background

Depression, anxiety, and stress are among the primary causes of disease rates worldwide [22] and are the most prevalent mental health problems in the USA [23]. Each is associated with significant economic, personal, intrapersonal, and societal losses including lower quality of life and increased mortality [8, 35, 37].

Depression and anxiety are also the most prevalent mental health problems among the student population [10, 34]. In the 2010 American College Health Survey, 48 % of college and university students reported feeling overwhelming anxiety at least once in the previous year. Thirty-one percent reported feeling so depressed that it was difficult to function at least once in the previous 12 months [2]. More recently, university counseling and mental health services have reported an increase in the number of clients seeking services and in yearly visits [5].

The college years can be a highly stressful time in students’ lives. This is particularly true of the period of transition to college as students are learning to cope with increased academic pressures [27, 48]. Students are at a developmental stage when newfound stressors can promote the onset of mental health difficulties [12, 46, 47]. In addition to academic stress, international students can experience significant sociocultural adjustment demands [19].

Young adults between 17 and 25 years of age are reported to be at greater risk of developing a serious mental illness than individuals in other age groups [51]. Although early diagnosis of mental disorders can be difficult, delayed diagnosis can often lead to treatment resistance and poorer longer-term outcomes [47, 53]. In addition, underachievement or failure at this point in life can cause long-term setbacks to individuals’ self-esteem and future progress [21, 47].

### Treating depression, anxiety, and stress

Depression, anxiety, and stress disorders can each be treated effectively using medications; however, after completing a course of this type of treatment, the chances of relapse are high, and equally successful psychological therapies are available [36]. Of these, cognitive-behavior therapy (CBT) is the most widely researched and CBT is recognized as the leading choice of treatment for depression and anxiety in terms of post-treatment improvements, maintaining progress, and preventing relapse [18, 30]. CBT also is effective for stress management [44]. CBT is comprised of a variety of cognitive and behavioral approaches, each concerned with changing distressing thoughts and beliefs. Treatment is often comprised of self-monitoring and thought recording, behavioral activation, cognitive restructuring, and exposure [11].

### Access to treatment

On a global scale, a significant number of individuals in need of psychological treatment receive no medical diagnosis nor do they seek treatment [1]. The worldwide treatment gap between those needing treatment for depression compared to those receiving treatment has been estimated at 56 %; the gap in treatment for anxiety disorders has been estimated at 46 % [25]. Several factors prevent people from accessing treatment, such as waiting lists, lack of motivation, negative attitudes about treatment, and costs [25, 29]. Among those willing to seek treatment, many encounter a lack of trained professionals or are placed on a waiting list [47]. Although students can often obtain mental health services on campus, many campuses have limited services or have wait lists for services because of the high demand [24]. A survey of US students with mental health problems revealed that common barriers to seeking services, even if they were available, were stigma, lack of time/accessibility, and wait lists [15]. Internet-delivered interventions are one way to address these issues.

### Internet-delivered CBT (iCBT)

Internet-delivered CBT programs are specially designed for the treatment of specific disorders and can be either clinician-guided or self-administered. When such interventions are used in the UK, 6–8 sessions are the recommended length of treatment [31–33]. Many studies provide support for the utility and effectiveness of internet-delivered treatments for depression, anxiety, and stress [4, 10, 38]. Those with the additional feature of human support tend to produce better results [38].

Several studies also have assessed the efficacy of online interventions for depression, stress, and anxiety for college students. Recent reviews of these studies have found evidence for the efficacy of these online interventions [9, 14]. However, most of these studies do not assess the potential for change of the interventions in the context of existing service delivery systems. Rather, many are RCTs assessing efficacy in convenience samples of students (e.g., Psychology majors). The current trial seeks to investigate the acceptability and potential for change of internet-delivered interventions among university students seeking services and the procedures needed to incorporate these interventions into mental health delivery in US universities. Additionally, it hopes to support and extend existing empirical evidence for SilverCloud programs [40–42].

### Objectives of the trial

The aim of this study is to test the acceptability of internet-delivered treatment for depression, anxiety, and stress in university students. The trial will investigate the feasibility of recruiting students (in relation to numbers screened, numbers recruited, and level of adherence) to receive internet-delivered interventions for the treatment of depression, anxiety, and stress and establish the acceptability of the interventions to the students at University of Minnesota.

Acceptability of the intervention to clients will be assessed using data on usage and engagement with the intervention (e.g., percentage of participants completing each module, average number of logins, average time spent per session, and total time spent on the program). These data are acquired through the online SilverCloud system. Satisfaction will be assessed through the use of a post-intervention questionnaire on satisfaction with accessing and using an online delivery format for treatment. At 3 month-follow-up, all 105 participants will be contacted by email and invited to complete a semi-structured interview about their perceptions, attitudes, and experiences of the intervention. The first 25 to respond will be asked to complete the interview. The interview sample will include participants who dropped out of the intervention.

The trial also hopes to establish the potential for change and to establish initial estimates of the effectiveness of these online interventions for students in terms of within-group effect sizes associated with changes in depression, anxiety, and stress from baseline to 8 week and 3 months follow-ups. These data will be used to estimate the sample size for a future trial to ensure that the study is sufficiently powered. A conservative estimate using the 90 % upper confidence limit will be used to inform the sample size calculation of the future RCT.

## Method/design 

### Study design

The study is an open trial of internet-delivered CBT treatment of depression, anxiety, and stress. After completing screening questionnaires, participants will be able to choose one of three online CBT interventions (Space from Depression, Space from Anxiety, and Space from Stress). The study protocol was approved by the University of Minnesota Institutional Review Board (March 18, 2015) (code number: 1503S64741).

### Recruitment procedure

Participants will be recruited through three units at the University of Minnesota-Twin Cities, a public research university located in Minneapolis and St. Paul, Minnesota. The first unit is Student Counseling Services (SCS) which provides free short-term counseling to undergraduate and graduate students. The second is Boynton Mental Health Clinic (BMHC) which also provides short-term psychological services to full-time students at the University of Minnesota. Unlike SCS, BMHC also has psychiatrists on staff and is affiliated with the university health service. Finally, we will recruit participants through the International Student and Scholar Services (ISSS) office which provides counseling to foreign national students on campus.

The SilverCloud programs will be advertised to students through the three participating offices on campus via staff referrals, brochures, flyers, email announcements, and web sites and through the general student mental health web site. Regardless of how they learn about the programs, students can sign up online to get access to the programs. If a student expresses interest, they will be emailed a link to a web site with more information about the programs and the study. Interested students will sign the consent form and complete screening measures online and will be given feedback on their scores. This feedback consists of informing students of their overall score for each measure and whether their score indicates mild, moderate or severe depression, anxiety, or stress. This will allow them to make an informed decision on choosing one of the three programs. They will then be referred to a supporter from within the service where they learned about the study. Participants’ post outcome measures will be gathered 8 weeks after their initial log in or activation of the program and 3 months later (5 months after initial log in).

A second recruitment phase will be conducted at 3-month follow-up. All 105 participants will be contacted by email and invited to complete a semi-structured interview about their perceptions, attitudes, and experiences of the intervention. From those that respond expressing an interest, the first 25 will be selected to complete a semi-structured interview.

### Sample size

**Fig. 1:**
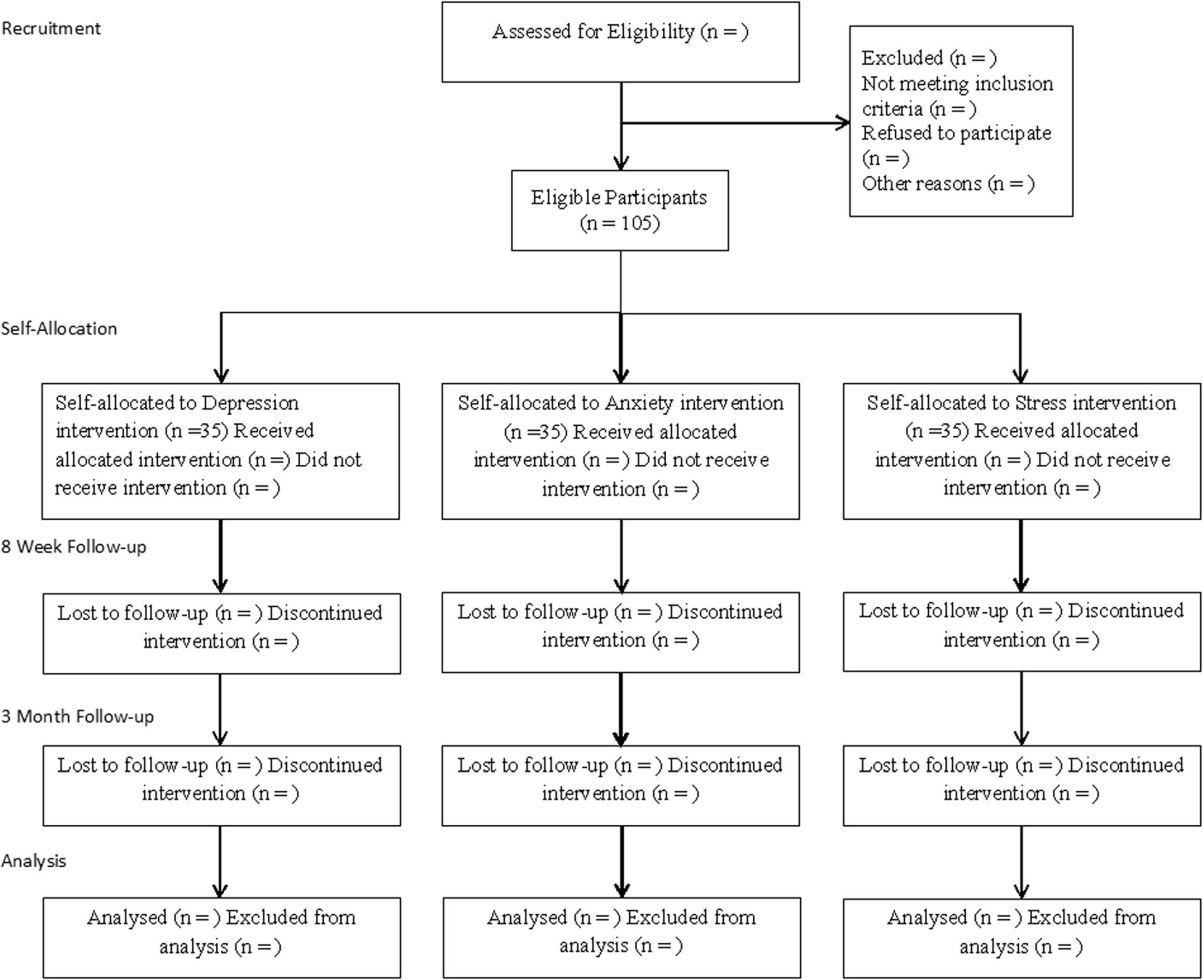
Flowchart of the study CONSORT

A sample size of 35 participants per group is the standard expectation for feasibility studies [52]. This sample size will allow us to estimate the standard deviation of the symptom outcome measures (for the proposed RCT). We thus aim to recruit 105 participants in total across the three trial arms (see Fig. 1). In addition, a sample of between 10 and 15 people would be suitable to reach saturation through analysis of data collected in semi-structured interviews [16].

### Eligibility criteria

All registered students at the University of Minnesota will be potentially eligible to participate. Eligibility criteria include being at least 18 years of age and self-reporting mild to severe symptoms of depression (5–27+) on the Patient Health Questionnaire-9 (PHQ-9; [26, 50]), mild to severe symptoms of anxiety (5–21+) on the Generalized Anxiety Disorder (GAD-7; [49]), or mild to severe symptoms of stress (15–34+) on the stress subscale of the Depression, Anxiety and Stress scale-21 (DASS-21; [28]). Students who score below the cutoff score on a given measure may still use the program module related to that measure, although their data will not be included in the analyses. Participants attending face-to-face individual or group counseling will be excluded. This includes all types of psychological therapy such as CBT. At baseline and 3 month assessments, participants will be asked if they have accessed any other form of psychological intervention throughout the time frame of the trial.

Students who score in the “red zone” in terms of risk of self-harm on the screening questions routinely used at SCS (i.e., the Behavioral Health Measure; [6]) will not be referred to the study. Students who score greater than 0 on the PHQ-9 self-harm item in the online screening will be automatically alerted that a counselor will try to reach them. They will be recommended to seek help from BMHC or UCCS. They will be telephoned within 1 working day and contacted by email if they cannot be reached by phone. There is also a “Help” button available on the site which can be accessed at any time and which directs students to a list of counseling resources and health services on campus.

### Interventions

#### Internet-delivered cognitive-behavior therapy (iCBT) programs

Space from Depression, Space from Anxiety, and Space from Stress are eight-module online CBT interventions, delivered on a Web 2.0 platform using media-rich interactive content (see Table 1 for a description of the Space from Depression program). Specifically, Space from Depression focuses on cognitive and behavioral components including self-monitoring and thought recording, behavioral activation, cognitive restructuring, and challenging core beliefs. Space from Anxiety includes problem solving strategies, collaborative empiricism, empowerment, and mindfulness. Space from Stress is primarily aimed at boosting positive well-being, addressing stress in university settings, and encouraging users to build a more balanced, enjoyable, and meaningful life. The content of the program modules follows evidence-based CBT principles. Each program is structured and incorporates introductory quizzes, videos, informational content, and interactive activities, as well as homework suggestions and summaries. In addition, personal stories and accounts from other users are presented. Participants will be encouraged to complete one module per week over 8 weeks [32], with weekly reviews provided by their supporter (10–15 min).

**Table 1:** Overview of Space from Anxiety program

Module name	Brief description
Welcome to SilverCloud	Introduction to the program to orient users to the program’s structure and content. Invites users to consider their expectations of the program.
Getting Started	Outlines the basic principles of CBT and provides information about anxiety. Users are encouraged to begin to chart their own difficulties with anxiety.
Understanding Feelings	Focus on developing emotional literacy and understanding the connection between thoughts, feelings and behaviors. Explore different aspects of emotions, physical body reactions, anxiety and behavior, and the impact of lifestyle choices and purposeful relaxation on these domains.
Facing Your Fears	Highlights the importance of defining fears and introduces graded exposure strategy. Users can define a fear hierarchy and carry out experiments to gradually face their fears and reduce their anxiety. Encourages the user to accept their feelings and embrace uncertainty.
Spotting Thoughts	Identify anxious thoughts, and notice how they are typically accepted without question. Explore ways to disempower thoughts through balanced acceptance. Ideas are presented about how worry can perpetuate anxiety, and how some distraction strategies can be beneficial.
Challenging Thoughts	Challenge distorted thinking errors or patterns, and outlines helpful ways of thinking. Ideas around the identification of negative hot thoughts and the development of alternatives are also presented.
Bringing It All Together	Reflect on any change in perspectives that may have occurred and to plan for staying well. The important role of social support is outlined and set backs are normalized.

### SilverCloud platform

The SilverCloud platform was developed in Ireland by a multi-disciplinary team of clinical, design, and development professionals under the company name of SilverCloud Health. Delivered through the SilverCloud platform, the programs for the treatment of depression, anxiety, and stress employ several innovative engagement strategies for improving the user experience. These are divided into several categories: personal, interactive, supportive, and social.

#### Personal

Users have their own secure homepage and can fill in a profile with basic information about themselves. Along with establishing a sense of ownership, this information allows the supporter to provide more personal feedback. The homepage is intended to provide a reflective space; users can document their thoughts and feelings, and these can be elaborated on within the journal application, which also acts as the vehicle for therapeutic writing exercises. Users have actions suggested to them, and as they complete program modules, their achievements are noted. Users are free to access the modules in any order they wish, contributing to a sense of empowerment. Along with the central content, a range of satellite applications are provided, such as a goal-setting application that can be used independently of the program content. Applications are released as the user completes the modules, with the intention of maintaining engagement by introducing new features over time and not overwhelming the user initially.

#### Interactive

The program includes a number of interactive elements and graphical exercises aimed at engaging users with the therapeutic content (e.g., reflecting on their own thinking style). Users also have the ability to comment on content within the programs. Both exercises and comments can be explicitly shared with the supporter. The user is provided with immediate feedback wherever possible; for example, when a mood chart is completed, the application item is graphically updated on the home page.

#### Supportive

All users have an assigned supporter who provides weekly reviews of their progress in the program. This support is asynchronous; the supporter sets a date to review a user’s progress and does not provide feedback, support or contact outside this time. The supporter can support multiple users and can review the work of all their users within an allocated time period. Such asynchronous online contact may be logistically easier to implement for many services compared to, for example, motivational interviewing and telephone support. Users are encouraged to share their content (such as completed exercises and comments) with their supporter. This enables the supporter to respond in a more personal way and provide guidance and encouragement to keep using the program. Adherence information is also available to the supporters so they can keep track of the users’ progress. Because this information is personally sensitive, users can see the supporter’s view of their data. Making the visibility of user data to the supporter more transparent, and giving the user the ability to change the sharing status of data, provides the user with a greater sense of control.

#### Social

As one step to making the programs social, the user can see anonymous indications of other people in the system. The intention is to reassure users that they are not alone in experiencing difficulties and that many other people have experienced similar problems and overcome them. Users can respond to content by indicating that they “like” it, and can see how many other people liked it, helping to reduce the sense of isolation. Introducing contact with other users within any online system raises a number of ethical concerns regarding the possibility for unhelpful or negative content or communications. Thus, other more detailed shared content (such as tips and ideas) is subject to supporter moderation.

### Supporter’s role during treatment

Each participant will be assigned a supporter who will monitor the participant’s progress throughout the trial. Supporters will be professional staff at SCS, BMHC, or ISSS. Once participants choose a program, there will be a message from their supporter at their first login. This message welcomes them to the program, highlights aspects of the program, and encourages them in the use of the program. Each week, the supporters will log in and review participants’ progress, leaving feedback for them and responding to the work they have completed. The basic share level allows supporters to view users’ goals for the week, key messages, and progress points. If users wish to share more with their supporter, they can share journal entries. Participating supporters will receive training in the program and how to deliver feedback. Each supporter will provide post-session feedback of between 10 and 15 min per participant per session for their assigned participants. No adherence measure was developed to control for variance in the feedback provided as this natural variance between supporters reflects the expected variance in feedback found in a naturalistic setting.

### Measures

Acceptability data will include information acquired through the online SilverCloud system including data on usage and engagement. In addition, the satisfaction with treatment (SAT) measure will be completed at post-treatment, and finally, data from semi-structured interviews with participants will inform acceptability.

The Patient Health Questionnaire-9 (PHQ-9), the Generalized Anxiety Disorder-7 (GAD-7), the stress subscale of Depression, Anxiety, Stress Scale-21 (DASS-21), and a sociodemographic and history questionnaire will be completed at screening. The following measures, PHQ-9, GAD-7, and stress subscale of DASS-21 will be completed again 8 weeks after their initial log in or activation of the program and 3 months later. All data will be obtained through online self-reported measures.

### Sociodemographic information

The sociodemographic information and history questionnaire is based on the history questionnaire used in an earlier study [43]. It will be developed for the present study and will collect basic demographic information on the participants (e.g., age and gender) as well as information on any previous diagnosis of depression, anxiety, and stress disorders and on the length of time those symptoms were experienced; participants’ experiences of counseling, therapy, and medication for depression, anxiety, and stress; previous diagnosis of an organic mental health disorder such as schizophrenia, psychosis, or bipolar disorder; and items related to the presence of psychosis, alcohol and drug misuse, and/or any recent medical diagnosis.

### Symptom outcome measures

The PHQ-9 [26, 50] is a 9-item self-report measure of depression that has been widely used in screening, primary care, and research. The PHQ-9 items reflect the diagnostic criteria for depression outlined in the *Diagnostic and Statistical Manual of Mental Disorders, Fourth Edition–Text Revision* (DSM–IV–TR) [3]. Items are rated on a 0 to 3 scale. Summed scores range from 0 to 27; higher scores reflect a greater severity of depressive symptoms [26, 50].

The GAD-7 [49] comprises 7 items measuring symptoms and severity of GAD based on the DSM-IV diagnostic criteria for GAD. The GAD-7 has good internal consistency (.89) and good convergent validity with other anxiety scales. Items are rated on a 0 to 3 scale. Summed scores range from 0 to 21; higher scores indicate greater severity of symptoms [49].

Stress subscale of Depression, Anxiety and Stress scale-21 (DASS-21). The DASS-21 [28] is an abbreviated version of the original 42-item DASS. It is composed of three 7-item subscales measuring symptoms of depression, anxiety, and stress. Participants will be asked to rate the degree to which they endorse each of the seven stress items on a 4-point scale (0 to 3). Summed scores range from 0 to 21; higher scores indicate greater severity of symptoms. Scores on the DASS-21 have been found to be valid measures of depression, anxiety, and stress in non-clinical samples [17].

### Acceptability

Acceptability of the intervention will be assessed using data acquired through the online SilverCloud system on usage and engagement with the intervention, including percentage of participants completing each module, average number of log ins, average time spent per session, and total time spent on the program.

We will also gather descriptive data on the feasibility of the recruitment process, such as numbers who expressed interest in participating, numbers who received links to the programs, and, of those, how many completed screening measures and how many used the program. Additionally, we will gather data on numbers recruited at each site and numbers of students selecting each of the three programs. These data will help to develop realistic estimates of enrollment accrual and attrition rates.

### Satisfaction with treatment

The satisfaction with treatment (SAT) questionnaire [40] includes nine quantitative questions regarding satisfaction with accessing treatment online (e.g., How did this online treatment compare to previous treatments? 0 = “much better” to 4 = “not at all good”). The satisfaction measure also contains two qualitative questions asking participants to describe what they most liked and least liked about the online treatment.

### Semi-structured interviews

Semi-structured interviews will include a schedule of questions derived from the literature that will explore the participants’ perceptions, attitudes, and experiences of the intervention. Interviews will be conducted by doctoral students via telephone and will last no more than 60 min.

### Ethical considerations

Information made available to all prospective participants will inform them of exactly what is involved in participating, including the objectives of the trial. Participants will know that their involvement is voluntary and they can withdraw their participation at any time without prejudice. Informed consent will be collected online via checking a box indicating that they consent to participate.

### Planned statistical analysis

Acceptability data in terms of usage and engagement will be assessed by analyzing the percentage of participants completing each module, the average number of logins, the average time spent per session, and total time spent on each of the three programs. As mentioned, feasibility will be assessed by analyzing the number of participants recruited at each site and the number of students selecting each of the three programs overall and at each site.

Satisfaction data will be analyzed using the descriptive and interpretative framework described by Elliott and Timulak [13].

Descriptive data will be presented from the semi-structured interviews. The transcriptions will be analyzed thematically using a pre-determined framework derived from the interview schedule and adapted and revised based on participant responses [45].

The outcome data will be treated as preliminary and interpreted with caution. Statistical analysis will be used to describe the characteristics of the sample and to provide initial estimates of parameters needed to inform the design of a future RCT. To assess whether change can be seen in the three programs, within group effect sizes (Cohen’s *d*) assessing change from baseline to 8 weeks and 3 months will be calculated. For Cohen’s *d*, an effect size of 0.2 to 0.3 can be considered a small effect, around 0.5 a medium effect, and 0.8 upwards a large effect [7]. A repeated measure ANOVA will be used to analyze the differences in the symptom outcomes across time points. An estimation of recovery will be made by identifying the number of participants completing each program who demonstrated a reduction of 50 % from pre-treatment PHQ-9, GAD-7, and DASS-21 stress subscale scores [39]. In addition, we will calculate the number of reliably changed and recovered participants using Jacobson and Truax’s [20] criteria.

## Discussion

This study seeks to assess the feasibility, acceptability, and proof of concept of internet-delivered treatment for depression, anxiety, and stress for university students. The study will contribute to understanding how low-intensity internet-delivered programs can be integrated into service delivery in university settings. Thus, the study will not only contribute to the already established work on online psychological treatments worldwide but will extend the limited research assessing the effectiveness of online psychological treatments in realistic service delivery settings including existing empirical evidence for SilverCloud programs [40–42]. Because participants will be recruited through three units at the University of Minnesota, including foreign national students from the International Student and Scholar Services, it is anticipated that the study will provide the researchers and service personnel with unique insight into students’ interest in and engagement with the programs, their user experience, and their satisfaction with the online delivery format. Any change in outcomes will be treated as preliminary and will be used to inform the methods for a future randomized trial of efficacy.

The symptom outcome measures (PHQ-9, GAD-7, DASS-21 stress subscale) that will assess depression, anxiety, and stress are well-established measures and each has been used in previous trials of internet-delivered and face-to-face treatments [42]. The acceptability and satisfaction measures will contribute information regarding what participants find satisfying with online treatments [40, 41]. Feasibility data will inform the procedures of a future trial (e.g., which programs to offer).

### Trial status

Participant recruitment commenced in October 2015 and is ongoing. Numbers to date are 63.

## References

[1] Alegria M, Kessler RC, Bijl R, Lin E, Heeringa SG, Takeuchi DT, Andrews G (2000). Comparing mental health service use data across countries. Unmet need in mental health service delivery.

[2] American College Health Association (2010). National college health assessment reference group executive summary.

[3] American Psychiatric Association (2000). Diagnostic and statistical manual of mental disorders: DSM-IV-TR.

[4] Andersson G, Cuijpers P (2009). Internet-based and other computerized psychological treatments for adult depression: a meta-analysis. Cogn Behav Ther.

[5] Association for University and College Counseling Center Directors (2014). The association for university and college counseling center directors annual survey.

[6] Bryan C, Blount T, Kanzler K, Morrow C, Corso K, Corso M, Ray-Sannerud B (2014). Reliability and normative data for the Behavioral Health Measure (BHM) in primary care behavioral health settings. Fam Syst Health.

[7] Cohen J (1988). Statistical power analysis for the behavioral sciences.

[8] Cuijpers P, Smit F, Oostenbrink J, de Graaf R, ten Have M, Beekman A (2007). Economic costs of minor depression: a population-based study. Acta Psychiatr Scand.

[9] Davies EB, Morriss R, Glazebrook C (2014). Computer-delivered and web-based interventions to improve depression, anxiety, and psychological well-being of university students: a systematic review and meta-analysis. J Med Internet Res.

[10] Day V, McGrath PJ, Wojtowicz M (2013). Internet-based guided self-help for university students with anxiety, depression and stress: a randomized controlled clinical trial. Behav Res Ther.

[11] Donohue EO, Fisher JE (2012). Cognitive Behavior Therapy: Core principles and practice.

[12] Dyson R, Renk K (2006). Freshmen adaptation to university life: depressive symptoms, stress, and coping. J Clin Psychol.

[13] Elliott R, Timulak L, Miles J, Gilbert P (2005). Descriptive and interpretative approaches to qualitative research. A handbook of research methods in clinical and health psychology.

[14] Farrer L, Gulliver A, Chan JK, Batterham PJ, Reynolds J, Calear A, Griffiths KM (2013). Technology-based interventions for mental health in tertiary students: systematic review. J Med Internet Res.

[15] Gruttadaro N, Crudo D. (2012). College students speak: A survey report on mental health. Retrieved from National Alliance on Mental Illness website: https://www.nami.org/About-NAMI/Publications-Reports/Survey-Reports/College-Students-Speak_A-Survey-Report-on-Mental-H.pdf.

[16] Guest G, Bunce A, Johnson L (2006). How many interviews are enough? An experiment with data saturation and variability. Field Methods.

[17] Henry JD, Crawford JR (2004). The sort-form version of the Depression Anxiety Stress Scales (DASS-21): construct validity and normative data in a large nonclinical sample. Br J Clin Psychol.

[18] Hersen M, Sturmey P (2012). Handbook of evidence-based practice in clinical psychology, volume 2, adult disorders (Vol. 2).

[19] Hirai R, Frazier P, Syed M (2015). Psychological and sociocultural adjustment of international students: trajectories and predictors. J Couns Psychol.

[20] Jacobson NS, Truax P (1991). Clinical significance: a statistical approach to defining meaningful change in psychotherapy research. J Consult Clin Psychol.

[21] Johnson EM, Coles ME (2013). Failure and delay in treatment-seeking across anxiety disorders. Community Mental Health Journal.

[22] Kessler RC, Aguilar-Gaxiola S, Alonso J, Chatterji S, Lee S, Ormel J, Wang PS (2009). The global burden of mental disorders: an update from the WHO world mental health (WMH) surveys. Epidemiol Psichiatr Soc.

[23] Kessler RC, Berglund P, Demler O, Jin R, Merikangas KR, Walters EE (2005). Lifetime prevalence and age-of-onset distributions of DSM-IV disorders in the National Comorbidity Survey Replication. Arch Gen Psychiatry.

[24] Kitzrow MA (2003). The mental health needs of today’s college students: challenges and recommendations. NASPA J.

[25] Kohn R, Saxena S, Levav I, Saraceno B (2004). The treatment gap in mental health care. Bull World Health Organ.

[26] Kroenke K, Spitzer RL, Williams JBW (2001). The PHQ-9 validity of a brief depression severity measure. J Gen Intern Med.

[27] Lee C, Dickson D, Conley C, Holmbeck G (2014). A closer look at self-esteem, perceived social support, and coping strategy: a prospective study of depressive symptomatology across the transition to college. J Soc Clin Psychol.

[28] Lovibond SH, Lovibond PF (1995). Manual for the depression anxiety stress scales.

[29] Mohr DC, Ho J, Duffecy J, Baron KG, Lehman KA, Jin L, Reifler D (2010). Perceived barriers to psychological treatments and their relationship to depression. J Clin Psychol.

[30] Nathan PE, Gorman JM (2007). A guide to treatments that work (treatments that work).

[31] National Institute for Health and Clinical Excellence (2006). Computerised cognitive behaviour therapy for depression and anxiety. Technology Appraisal 97.

[32] National Institute for Health and Clinical Excellence (2009). The treatment and management of depression in adults.

[33] National Institute for Health and Clinical Excellence (NICE) (2011). Commissioning stepped care for people with common mental health disorders.

[34] Newton-Taylor B, Adlaf E, Gliksman L, Demers A (2001). The prevalence of elevated psychological distress among Canadian undergraduates: findings from the 1998 Canadian campus survey. J Am Coll Heal.

[35] Rapaport MH, Clary C, Fayyad R, Endicott J (2005). Quality-of-life impairment in depressive and anxiety disorders. Am J Psychiatry.

[36] Reynolds CF, Beekman ATF, Sijbrandij EM, Cuijpers P, Koole SL, Andersson G (2014). Adding psychotherapy to antidepressant medication in depression and anxiety disorders: a meta-analysis. World Psychiatry.

[37] Richards D (2011). Prevalence and clinical course of depression: a review. Clin Psychol Rev.

[38] Richards D, Richardson T (2012). Computer-based psychological treatments for depression: a systematic review and meta-analysis. Clin Psychol Rev.

[39] Richards DA, Suckling R (2009). Improving access to psychological therapies: phase IV prospective cohort study. Br J Clin Psychol.

[40] Richards D, Timulak L (2012). Satisfaction with therapist-delivered vs. self-administered online cognitive behavioural treatments for depression symptoms in college students. Br J Guid Couns.

[41] Richards D, Timulak L (2012). Client-identified helpful and hindering events in therapist delivered vs. self-administered online cognitive-behavioural treatments for depression in college students. Couns Psychol Q.

[42] Richards D, Timulak L, Doherty G, Sharry J, Bligh J, Colla A (2014). Internet-delivered treatment for depression: its potential as a low-intensity community intervention for adults with depression: Protocol for a randomized controlled trial. BMC Psychiatry.

[43] Richards D, Timulak L, Hevey D (2013). A comparison of two online cognitive-behavioural interventions for symptoms of depression in a student population: the role of therapist responsiveness. Couns Psychother Res.

[44] Richardson K, Rothstein H (2008). Effects of occupational stress management intervention programs: a meta-analysis. J Occup Health Psychol.

[45] Ritchie J, Lewis J (2003). Qualitative research practice: a guide for social science students and researchers.

[46] Royal College of Psychiatrists (2003). The mental health of students in higher education.

[47] Royal College of Psychiatrists (2011). Mental health of students in higher education.

[48] Rutter M, Sroufe LA (2000). Developmental psychopathology: concepts and challenges. Dev Psychopathol.

[49] Spitzer RL, Kroenke K, Williams JW, Löwe B (2006). A brief measure for assessing generalized anxiety disorder: the GAD-7. Arch Intern Med.

[50] Spitzer R, Kroenke K, Williams J (1999). Validation and utility of a self-report version of PRIME-MD: the PHQ primary care study. J Am Med Assoc.

[51] Substance Abuse and Mental Health Services Administration. (2009). Results from the 2009 national survey on drug use and health: Volume I. Summary of national findings. Retrieved from http://archive.samhsa.gov/data/NSDUH/2k9NSDUH/2k9Results.htm.

[52] Teare MD, Dimairo M, Shephard N, Hayman A, Whitehead A, Walters SJ (2014). Sample size requirements to estimate key design parameters from external pilot randomised controlled trials: a simulation study. Trials.

[53] Wang PS, Berglund PA, Olfson M, Kessler RC (2004). Delays in initial treatment contact after first onset of a mental disorder. Health Serv Res.

